# Haploidentical Stem Cell Transplantation With Dual Source of Cells and Post‐Transplant Cyclophosphamide

**DOI:** 10.1002/cam4.70541

**Published:** 2025-01-31

**Authors:** Ana Marcela Rojas Fonseca‐Hial, Katya Parisio, Jose Salvador Rodrigues de Oliveira

**Affiliations:** ^1^ Transplante de Medula Ossea Section Federal University of Sao Paulo—UNIFESP Sao Paulo Brazil; ^2^ Transplante de Medula Ossea Section Hospital Santa Marcelina Sao Paulo Brazil

**Keywords:** allogeneic, dual source of cells, haploidentical, post‐transplant cyclophosphamide, reduced intensity conditioning

## Abstract

**Background:**

Dual sources of cells (DSC) with peripheral blood stem cell apheresis (PBSC) and surgical bone marrow (BM) for haploidentical hematopoietic cell transplantation (Hid‐HCT) are used in China and some Asian countries. The experience of the Baltimore group for haploidentical transplant with post‐transplant cyclophosphamide (PT‐Cy) and reduced‐intensity‐conditioning (RIC) regimen used BM as a source of hematopoietic stem cells.

**Methods:**

We retrospectively analyzed 64 Hid‐HCT with DSC and PT‐Cy, RIC (*n* = 57), or myeloablative‐conditioning (MAC) (*n* = 7), from two public health Brazilian centers, with a median follow‐up of 23.3 months (6.7–45.4).

**Results:**

The 49 malignant patients were 27/46 (58.7%) beyond the first remission or with no complete response, and three patients did not complete disease status evaluation before transplant. Eight of 62 patients (12.9%) had grade 2 or more Hematopoietic Cell Transplantation‐Specific Comorbidity Index (HCT‐CI), and two patients had no HCT‐CI classified. Cytomegalovirus (CMV) viremia occurred in 26 of 57 (45.6%). The cumulative incidence of 100‐day grade III‐IV acute GVHD was 12.3% (7/57), with a 95% confidence interval (CI) of 3.8% and 20.8%, and 2‐year moderate or severe chronic GVHD was 21.1% (11/52; 95% CI, 10.1%–32.3%). The 2‐year relapses were 24.5% for malignant disease (12/49; 95% CI, 12.4%–36.5%). The 2‐year overall survival (OS) probability was 54.7% (35/64; 95% CI, 42.5%–66.9%). Benign diseases achieve 2‐year OS in 73.3% (11/15; 95% CI, 51%–95.7%) of the patients. The HCT‐CI were significant in multivariate analyses for DFS (*p* = 0.002) and OS in uni‐ and multivariate analyses (both *p* < 0.001). The number of CD34^+^ cells by apheresis collection was significant in multivariate analysis for DFS (*p* = 0.039).

**Conclusion:**

Hid‐HCT using PT‐Cy, DSC, and RIC is a safe option for benign and malignant diseases.

**Abbreviations**
ALL: acute lymphoblastic leukemia
AML: acute myeloid leukemia
ATG: antithymocyte globulin
BM: surgical bone marrow
CML: chronic myeloid leukemia
CMV: cytomegalovirus
CR1: first complete remission
CR2: second complete remission
CRGNB: carbapenem‐resistant gram‐negative bacilli
DSA: HLA donor specific antibodies
DSC: dual source of cells
DFS: disease free survival
G‐CSF: granulocyte‐colony stimulating factor
GvHD: graft‐versus‐host disease
HCT: hematopoietic cells transplantation
HCT‐CI: hematopoietic cell transplantation‐specific comorbidity index
Hid‐HCT: haploidentical hematopoietic cells transplantation
IRM: infection related mortality
KPC: Klebsiella pneumoniae carbapenemases
LFS: leukemia‐free survival
MAC: myeloablative conditioning
MDS: myelodysplasic syndromes
MRD: minimal residual disease
NRM: non‐relapse mortality
OS: overall survival
PBSC: peripheral blood stem cell
PT‐Cy: post‐transplant cyclophosphamide
SAA: severe aplastic anemia
TBI: total body irradiation
TNC: total nucleated cells
VOD/SOS: veno‐occlusive disease/sinusoidal obstruction syndrome
VRE: Vancomycin‐resistant enterococci

## 1 Introduction

Hid‐HCT has increased since 2012, overcoming the number of matched HLA‐identical HCT since 2020 [1]. In 2020, the Center for International Blood and Marrow Transplant Research (CIBMTR) reported that 72% of Hid‐HCT was from PBSC, the remaining from BM. In China, 99% of Hid‐HCT follow the Beijing Protocol: (1) granulocyte‐colony stimulating factor for all donors (G‐CSF); (2) intensive immune suppression; (3) anti‐human thymocyte immunoglobulin (ATG) for GVHD prophylaxis; and (4) the combination of G‐CSF primed PBSC and BM as hematopoietic stem cells sources [2, 3].

Zhao et al. in 2016 [4] compared Hid‐HCT with PBSC (*n* = 42) versus DSC (*n* = 168) in malignant disease beyond the first remission, using ATG for GVHD prophylaxis. There are no differences in non‐relapse mortality (NRM), relapse, engraftment, or acute and chronic GVHD, but a better 3‐year DFS and OS were disclosed for DSC, 59.9% versus 44.3% and 65% versus 54.2%, respectively. Wang et al. in 2015 [5] used the Beijing protocol with MAC and ATG for GVHD prophylaxis in 450 AML patients, Hid‐HCT (*n* = 231) or identical sibling transplants (*n* = 219). They disclosed similar results for OS, relapse, DFS, and NRM.

Luznik et al. in 2008 [6] evaluated the high‐dose PT‐Cy in Hid‐HCT to prevent engraftment rejection and GVHD. Engraftment rejection occurred in 13% of patients. The median neutrophil engraftment was 15 days. The Grades II–IV and III–IV acute GVHD by D + 200 were 34% and 6%, respectively. Extensive chronic GVHD at 1 year was 5% for two doses of 50 mg/kg and 25% for one dose (*p* = 0.05%). The probabilities of NRM at D + 100 and 1 year were 4% and 15%, and relapse was 51% and 58% for one or two doses (NS). OS and DFS were not statistically significant. This publication was the first one to enable the spread of Hid‐HSC elsewhere.

Bashey et al. evaluated 681 patients with hematologic malignancy who underwent haploidentical transplantation with PT‐Cy in the United States between 2009 and 2014 and received BM (*n* = 481) or PB (*n* = 190) grafts. Hematopoietic recovery was similar between BM and PB transplantations (28‐day neutrophil recovery, 88% vs. 93%, *p* = 0.07; 100‐day platelet recovery, 88% vs. 85%, *p* = 0.33). Risks of Grades 2–4 acute (hazard ratio [HR], 0.45; *p* = 0.001) and chronic (HR, 0.35; *p* = 0.001) graft‐versus‐host diseases in BM transplantations were lower than PB transplantations. There were no significant differences in overall survival by graft type (HR, 0.99; *p* = 0.98), with rates of 54% and 57% at 2 years after transplantation of BM and PB, respectively. There were no differences in non‐relapse mortality risks (HR, 0.92; *p* = 0.74), but relapse risks were higher after transplantation of BM (HR, 1.49; *p* = 0.009). Additional exploration confirmed that the higher relapse risk after the transplantation of BM was limited to patients with leukemia (HR, 1.73; *p* = 0.002) but not lymphoma (HR, 0.87; *p* = 0.64) [7].

The purpose of this study was to retrospectively analyze the results of the association of DSC based on Chinese experience and GVHD prophylaxis with PT‐Cy designed for Baltimore Protocol in two Brazilian centers. DSC in these institutions has been adopted since 2013 to achieve earlier engraftment, shorter hospitalization time, and fewer engraftment failures, despite the possibility of a higher incidence of acute and chronic GVHD. Due to the frailty of the assisted population, the priority was to perform RIC, leaving MAC only for patients with very advanced diseases.

## 2 Methods

### 2.1 Study Design

We analyzed 64 Hid‐HCT in two public hospitals in Brazil, Hospital Santa Marcelina and Hospital São Paulo UNIFESP, from July 2016 to October 2019. All patients who underwent Hid‐HCT with DSC in this period were invited to participate, and their medical records were reviewed. The ethics committees of these two institutions approved this study.

### 2.2 Conditioning Regimens

The conditioning most used for the Hid‐HCT was RIC, the Baltimore protocol [6]. This conditioning consists of fludarabine 30 mg/m^2^/day i.v. on Days −6 to −2, cyclophosphamide 14.5 mg/kg/day i.v. on Days −6 and −5, total‐body irradiation (TBI) 200 cGy, associated with PT‐Cy. The post‐transplant cyclophosphamide was applied on D + 3 and D + 4 at 50 mg/kg/day. In patients with hemoglobinopathies, thymoglobulin was associated with the conditioning, 5 mg/kg divided over 4 days until D‐1 [8]. The CSA was initiated on D + 5 at 2 mg/kg/day i.v., adjusted to achieve a therapeutic level of 100–300 ng/mL, and converted to oral form until discontinuation. If there was no active GVHD, CSA was tapered at D + 60 and discontinued at D + 120, except for benign diseases. Mycophenolate mofetil (MMF) was prescribed on D + 5 to D + 35, at a dose of 15 mg/kg three times daily.

Surveillance and GVHD were assessed thrice a week until D + 60 and twice a week until D + 100. Then, every 2 or 4 weeks, depending on progress. Blood cultures for infection were taken whenever patients had a fever or other signs of disease.

### 2.3 PBSC Mobilization and Collection

Donors were primed with G‐CSF, 5–10 mcg/kg/day for four or five consecutive days. On the fourth day, PBSCs were collected with a COBE Blood Cell Separator (Spectra LRS, COBE BCT Inc., Lakewood, CO, USA). The target of PBSC to be infused were 5–10 × 10^6^ cells/kg recipient weight. On the fifth day, bone marrow cells were harvested. The target total nucleated cell count (TNC) was 3–4 × 10^8^ cells/kg recipient weight. The fresh and unmanipulated bone marrow and PBSCs were infused on the day of surgical collection [2].

### 2.4 Antimicrobial Prophylaxis and Follow‐Up

Patients were admitted to HEPA‐filtered units and underwent anal swab examination to investigate colonization by multidrug‐resistant bacteria and CRGNB. Antimicrobial prophylaxis using ciprofloxacin, trimethoprim‐sulfamethoxazole, acyclovir, and fluconazole began at admission [9, 10, 11, 12]. This prophylaxis was maintained until the end of immunosuppression.

After engraftment, all patients were monitored for CMV infection with plasmatic DNA polymerase chain reaction (PCR) twice a week until Day 60, then once a week. Above 10,000 copies/mL, or a progressive increase, the preventive use of intravenous ganciclovir was started, up to two consecutive exams with a below 10,000/mL copies.

### 2.5 Statistical Analysis

The absolute and relative frequencies were calculated for the categorical variables; for numerical variables, the mean and standard derivation; and for the mean comparisons between more than two variables, the analysis of variance was performed. The normality determination in the variance distribution data was verified with the Kolmogorov–Smirnov test. Overall survival was defined as the time from HCT until the last medical appointment or death. Relapse was defined as the return of the malignant disease. Non‐relapse mortality (NRM) was characterized as death without evidence of relapse. Acute and chronic GVHD were determined as a reaction of donor immune cells against host tissues. The disease‐free survival (DFS) was defined as the time to the first event of relapse, death, or last medical appointment. Relapse, GVHD outcomes, and NRM have been calculated using the cumulative incidence function. Relapse incidence (RI) and NRM were mutually competing events. Death was a competing event of GVHD outcomes. Survival analyses were based on the Kaplan–Meier model and the effect of covariates on survival was based on the Cox model. Survival functions were estimated for each level of these variables and then compared using the Log Rank test (Mantel–Cox). In addition, univariate Cox models were fitted for all predictor variables, followed by a multivariate model. The significance level was set at 5%. Statistical analyses were performed using SPSS 20.0 and STATA 17 statistical software.

## 3 Results

From July 2016 to October 2019, there were 64 Hid‐HCT with DSC and PT‐Cy, Table 1. Malignant diseases were considered with advanced disease in 27/46 (58.7%), and three patients did not complete disease status evaluation before transplant. MRD was positive in 6/29 (20.7%) of acute leukemias, all lymphomas were in partial or worse response, and all cases of CML were refractory to 2nd‐generation TKI. Low, intermediate, high, and very high disease risk index (DRI) [13] were 2‐year OS 75%, 40.6%, 39.1%, and 46.8%, respectively. Of the benign diseases, the 10 patients with SAA had more than 10 red blood cell transfusions and a median diagnosis of 16.3 (3.8–164) months until Hid‐HCT. Sickle cell anemia patients had a median age of 17 [14, 15, 16, 17, 18, 19, 20, 21, 22, 23, 24] years and received multiple transfusions.

**TABLE 1:** Characteristics of patients.

Age years (range)		26 [3–66]
**Gender, *N*° (%)**		
Male		39 (61)
Female		25 (39)
**Diagnosis**		
**MALIGN DISEASES**		49/64 (76.5%)
*Acute leukemias*		32/49
Acute lymphocytic leukemia		17
	CR1, MRD neg	12
	CR2, MRD neg	2
	CR2 or CR3, MRD pos	3
Acute Myeloid Leukemia		13
	CR1, MRD neg	6
	CR1, MRD NA	3
	CR2 or CR3, MRD neg	2
	CR2 or CR3, MRD pos	2
Mixed Phenotype Acute Leukemia	CR2, MRD pos	1
Dendritic Cell Leukemia	CR1, MRD neg	1
*Chronic myeloid leukemia, TKI refractory*		7/49
Primary chronic phase		4
Blast phase		3
*Lymphoproliferative diseases*		9/49
Hodgkin lymphoma		7
	Partial response	3
	Stable disease	2
	Progressive disease	2
Non hodgkin lymphoma follicular	Partial response	1
T‐cell prolymphocytic leukemia	Morphologic response, MRD pos	1
*Myelodisplastic syndrome*		1/49
Multilineage dysplasia		1
**Disease Risk Index (DRI)**		
Low		8 (16.3%)
Intermediate		19 (38.7%)
High		14 (28.5%)
Very high		8 (16.3%)
**BENIGN DISEASES**		15/64 (23%)
*Bone marrow failure syndromes*		10/15
Severe aplastic anemia		9
PNH positive		6
PNH negative		3
Chronic acquired agranulocytosis		1
*Hereditary hemoglobinopathies*		5/15
Sickle cell anemia		4
Sickle‐beta thalassemia		1
**ABO compatibility**		
Matched		45 (70.3%)
Minor mismatch		8 (12.5%)
Major mismatch		10 (15.6%)
Bidirectional mismatch		1 (1.5%)
**Donor age years (range)**		29 [12–61]
**Donor‐recipient gender match**		
	Female–male	10 (15.6%)
	Male–male, female–female	41 (64%)
	Male–female	13 (20.3%)
**Donor‐recipient relationship**		
	Parent–child	17 (26.6%)
	Sibling–sibling	35 (54.6%)
	Child–parent	12 (18.7%)
**VRE and/or CRGNB colonization**		
	Positive	17/54 (31.5%)
	Negative	37/54 (68.5%)
	NA	10/64 (15.6%)
**DSA**		
	Positive (MFI > 2.000)	3/64 (4.7%)
	Negative	61/64 (95.3%)
**HCT‐CI**		
	0	31 (48.4%)
	1	23 (36%)
	2	5 (7.8%)
	≥ 3	3 (4.7%)
	NA	2 (3.1%)

Abbreviations: CR1, CR2 and CR3 = first, second and third complete response; CRGNB = carbapenem‐resistant gram‐negative bacilli ( *Escherichia coli* , *Klebsiella pneumoniae* , *Pseudomonas*, etc.); DSA = donor specific antibodies (HLA); MRD = minimal residual disease; NA = not available; PNH = paroxysmal nocturnal hemoglobinuria; VRE = vancomycin‐resistant enterococcus.

HLA donor‐specific antibodies (DSA) were negative in 61/64 (95.3%) patients, Table 1. One positive AML patient with DSA DQB1 title of 6592 had engraftment failure and autologous recovery at D + 34. Two other positive patients had neutrophil engraftment at D + 16 and D + 18. One DSA‐negative patient had engraftment failure and autologous recovery. Another engraftment failure occurred in a 27‐year‐old male SAA, who submitted the 1st Hid‐HSCT with Baltimore protocol using their mother's DSC and lost his engrafting at D + 39 after a pulmonary adenovirus and JC urinary infections. Then, he performed a successful 2nd Hid‐HCT with a DSC of his cousin, but this second transplant was excluded from this casuistic. Fifty‐seven of sixty‐four patients had engrafted.

RIC was performed in 57 transplants (89%), 56 patients with Baltimore conditioning. Fludarabine and melphalan [25] were performed in one patient with accelerated phase CML. MAC was performed in seven transplants (10.9%). Busulfan and cyclophosphamide [15, 16] were the conditioning for a CML resistant to a 2nd tyrosine kinase inhibitors (TKI) generation and in an ALL patient in the 1st negative MRD. Fludarabine and total body irradiation (TBI) 1200 cGy [17, 18] were used in two poor prognoses ALL patients. Fludarabine and busulfan [19] were used in chronic T‐prolymphocytic leukemia (CLL) in the 3rd morphological remission. Fludarabine, busulfan, and thiotepa [18, 20] were done for a 7‐year‐old boy with ALL at 3rd morphological remission. Etoposide nanoparticles and TBI 1200 cGy [21] were used in a patient with AML after MDS in 1st positive MRD with morphological remission, Table 2.

**TABLE 2:** Transplant procedure results.

**Conditioning**	
RIC	57/64 (89%)
FluCyTBI	56
FluMel	1
MAC	07/64 (10.9%)
BuCy	2
FluTBI	2
FluBu	1
FluBuTT	1
nanoVPTBI	1
**Infused hematopoietic cells**	
CD34^+^ cells × 10 (6)/kg (apheresis)	7.2 + − 2.9
MNCs × 10 (8)/kg (surgical harvest)	4.8 + − 1.6
**Neutrophil engraftment day**	16 [11–34]
Not available (death before engraftment) (7/64)	
**Hepatic veno‐occlusive disease**	5/64 (7.8%)
**Hospital discharge**	23 [17–50]
**Viral infection** ^a^	34/57 (59.6%)
CMV^a^	26/57 (45.6%)
**Fungal infection** ^a^	7/57 (12.3%)
**Acute GvHD III–IV** ^a^	7/57 (12.3%)
**Chronic GvHD moderate and severe** ^b^	11/52 (21.2%)
**Relapse 2y** ^c^	12/49 (24.5%)
**2y OS and DFS, malignant diseases**	49% and 44.9%
**2y OS, benign diseases**	73.3%

Abbreviations: DFS = disease‐free survival, FluBu = fludarabine and busulfan, FluCyTBI = fludarabine, cyclophosphamide and total body irradiation, FluMel = fludarabine and melphalan, FluTBI = fludarabine and TBI, GvHD = graft versus host disease, MAC = myeloablative conditioning, OS = Overall survival, RIC = reduced‐intensity conditioning.

^a^ Evaluated in 57 patients after engraftment.

^b^ Evaluated in 52 patients at 100 days after transplant.

^c^ Evaluated in 49 malignant diseases.

The number of infused PBSCs reduced the risk of disease progression. For every 1 × 10^6^ CD34 cells/kg increase, there was a 14% reduction in disease progression by multivariate analysis (*p* = 0.039).

SOS/VOD was observed in five patients (7.8%; 95% CI, 1.2%–14.4%), Table 2, all with advanced malignant diseases. Two had received myeloablative conditioning, the first died at D + 48 complicated with CMV infection, and the other had mild form, recovered from this complication, and is in complete remission after 17.5 months. Of the three patients with RIC, two relapsed and died at 10.5 and 21.6 months, and one recovered and is alive after 29 months.

Seven patients died before engraftment, 10.9% (95% CI, 3.3%–18.6%) all of infection, at a median of 13.4 days, ranging from 6 to 19. Six were previously colonized or infected. The seventh patient had a Hematopoietic Cell Transplantation‐Specific Comorbidity Index (HCT‐CI) 3 [22, 23], Table 3. HCT‐CI correlated with OS in univariate and multivariate analyses with *p* < 0.001. For every one‐point increase in HCT‐CI, there was an 82% increase in the risk of death, Table 4. The DFS for HCT‐CI was relevant only in multivariate analyses, with *p* = 0.002, Table 5.

**TABLE 3:** Death before engraftment.

Disease	Gender	Age (year)	HCT‐CI	Previous infections	Conditioning	Death cause	Death day
ALL	F	35	2	ESBL *Escherichia coli* urinary, VRE axilar and inguinal swab	FluCyTBI	*Pseudomonas aeruginosa* bloodstream; *Fusarium oxysporum* cutaneous.	19
HL	F	42	1	*Pseudomonas aeruginosa* bronchoalveolar, and KPC anal swab	FluCyTBI	KPC pneumonia	6
AML	M	46	5	*Pseudomonas aeruginosa* urinary, KPC and *Acinetobacter baumanii* anal swab	FluCyTBI	*Pseudomonas aeruginosa* bloodstream	15
ALL	M	52	1	KPC and VRE anal swab	BuCy	KPC bloodstream	10
SAA	M	20	1	VRE anal swab	FluCyTBI	*Pseudomonas aeruginosa* and CMV bloodstream	13
CML	F	53	3	No	FluCyTBI	Pulmonary sepsis	15
ALL	M	36	1	VRE swab anal	FluCyTBI	*Staphylococcus haemolyticus* bloodstream	16

Abbreviations: ALL = acute lymphocytic leukemia, AML = acute myeloid leukemia, CML = chronic myeloid leukemia, CML = chronic myeloid leukemia, HL = Hodgkin lymphoma, HL = Hodgkin lymphoma, KPC = *Klebsiella pneumoniae* carbapenemase, MRD = minimal residual disease, NA = not available, SAA = severe aplastic anemia, SAA = severe aplastic anemia, VRE = vancomycin‐resistant enterococcus.

**TABLE 4:** Cox regression for OS.

4.1 Univariate	Hazard ratio	*p*
Female (reference male)	1.57 (0.76–3.26)	0.226
Age (years)	1.01 (0.99–1.03)	0.375
HCT‐CI	1.89 (1.34–2.67)	< 0.001
Positive surveillance swab cultures (reference negative)	3.25 (1.53–6.90)	0.002
PBSC number (× 10^6^/kg)	0.91 (0.78–1.06)	0.210
BM number (×10^8^/kg)	0.90 (0.69–1.17)	0.445

4.2 Multivariate				
	Test 1		Test 2	
	Adjusted hazard ratio (95% CI)	*p*	Adjusted hazard ratio (95% CI)	*p*
HCT‐CI	1.71 (1.16–2.53)	0.007	1.89 (1.34–2.67)	< 0.001
Positive surveillance swab cultures (reference negative)	1.94 (0.85–4.41)	0.114		

*Note:* Proportional hazards test, test 1 chi (2) = 4.44, *p* = 0.109, and test 2 chi (1) = 1.79, *p* = 0.181.

**TABLE 5:** Cox regression for DFS.

5.1 Univariate	Hazard ratio (0.5)	*p*
Female (reference male)	1.60 (0.80–3.21)	0.183
Age (years)	1.01 (0.99–1.03)	0.357
HCT‐CI	1.61 (1.14–2.26)	0.006
Positive surveillance swab cultures (reference negative)	2.83 (1.37–5.87)	0.005
PBSC number (× 10^6^/kg)	0.89 (0.77–1.02)	0.093
BM number (× 10^8^/kg)	1.01 (0.78–1.31)	0.934

5.2 Multivariate				
	Model 1	*p*	Model 2	
	Adjusted hazard ratio (95% CI)		Adjusted hazard ratio (95% CI)	*p*
HCT‐CI	1.69 (1.12–2.55)	0.012	1.82 (1.25–2.65)	0.002
VRE and/or CRGNB colonization (positive × negative)	1.67 (0.69–4.09)	0.258	—	—
PBSC number (× 10^6^/kg)	0.86 (0.74–1.00)	0.052	0.86 (0.74–0.99)	0.039

*Note:* Proportional hazards test, test 1 Chi (2) = 6.46, *p* = 0.091, and test 2 Chi (2) = 3.21, *p* = 0.201.

Among the 57 patients who underwent engraftment, five (8.8%) died before D + 100, with a median of 1.6 months. Three infections deaths: two patients died from bloodstream *Klebsiella pneumoniae carbapenemase* (KPC) infections and were pre‐transplant infected, one from *Acinetobacter baumani* sepsis, and the other from CMV pulmonary infectious. And two deaths for acute GVHD were associated with Adenovirus infections. After D + 100, there were four infection deaths: two for pulmonary sepsis, one for KPC bloodstream, and the last for *Varicella‐zoster virus* (VZV), Table 6. CRGNB was significant in univariate analysis for DFS (*p* = 0.005) and OS (*p* = 0.002), Tables 4 and 5.

**TABLE 6:** Deaths concerning 100 days, excluded engraftment fail.

	Between engraftment and 100 days	After 100 days	Total
	*n* = 5/57 (8.8%)	*n* = 17/57 (29.8%)	*n* = 22/57 (38.6%)
**Age years (range)**	25 [5–56]	25.5 [3–66]	25 [3–66]
**Diagnosis**			
AML (*n* = 12)	1	6	7/12 (58.3%)
ALL (*n* = 14)	2	5	7/14 (50%)
CML (*n* = 6)	0	2	2/6 (33.3%)
HL (*n* = 6)	1	2	3/6 (50%)
SAA (*n* = 9)	1	1	2/9 (22.2%)
Sickle cell disease (*n* = 5)	0	1	1/5 (20%)
NHL, ABL, DCL, CLL, MDS (*n* = 1)	0	0	0
**Previous colonization**			
CRGNB Pos (*n* = 8)	0	3/17 (17.6%)	3/8 (37.5%)
CRGNB Neg (*n* = 36)	2/5 (40%)	7/17 (41.1%)	9/36 (25%)
VRE Pos (*n* = 03)	1/5 (20%)	1/17 (5.9%)	2/3 (66.7%)
ND (*n* = 10)	2/5 (40%)	6/17 (35.2%)	8/10 (80%)
**HCT‐CI**			
0 (*n* = 31)	1/5 (20%)	10/17 (58.8%)	11/31 (35.4%)
1 (*n* = 19)	2/5 (40%)	6/17 (35.3%)	8/19 (42.1%)
2 (*n* = 4)	1/5 (20%)	1/17 (5.9%)	2/4 (50%)
3 (*n* = 1)	1/5 (20%)	0	1/1 (100%)
ND (*n* = 2)	0	0	0
**Conditioning**			
RIC (*n* = 51)	4/5 (80%)	16/17 (94.1%)	20/51 (39.2%)
MAC (*n* = 6)	1/5 (20%)	1/17 (5.9%)	2/6 (33.3%)
**Cause of death**			
Infection	3/5 (60%)	4/17 (23.5%)	7/57 (12.2%)
GvHD	2/5 (40%)	2/17 (11.7%)	4/57 (7%)
Relapse^a^	0	11/17 (64.7%)	11/49 (22.4%)^a^

^a^ Evaluated at 49 patients with malignant diseases.

There was one case of tuberculosis (1.7%). He was transplanted with stable HL and relapsed at 12.6 months. Pulmonary tuberculosis was diagnosed at 14 months, and there is no more evidence of infection at 18.4 months.

There were five pulmonary invasive Aspergillosis. One after MAC died, and four after RIC were successfully treated. Preemptive treatment for CMV was done in 26/57 (45.6%). There were four (7%) VZV infections; two adenovirus infections (3.5%), and one urinary JC virus with engraftment lost.

Grades III–IV acute GVHD were observed in 12.3% (7/57; 95% CI, 3.8%–20.8%), one just after donor lymphocyte infusions (DLI). Two of the seven died of Grade IV acute GVHD on D + 48 and D + 93. Chronic GVHD was seen in 21.2% (11/52; 95% CI, 10.1%–32.3%) transplants after D + 100. The extensive form was observed in 3/52 (5.8%). Two extensive chronic GVHD patients died on D + 201 and 237, with hepatic and cutaneous involvements. The third patient presented severe sclerotic cutaneous and cholestatic liver injury.

**FIGURE 1:**
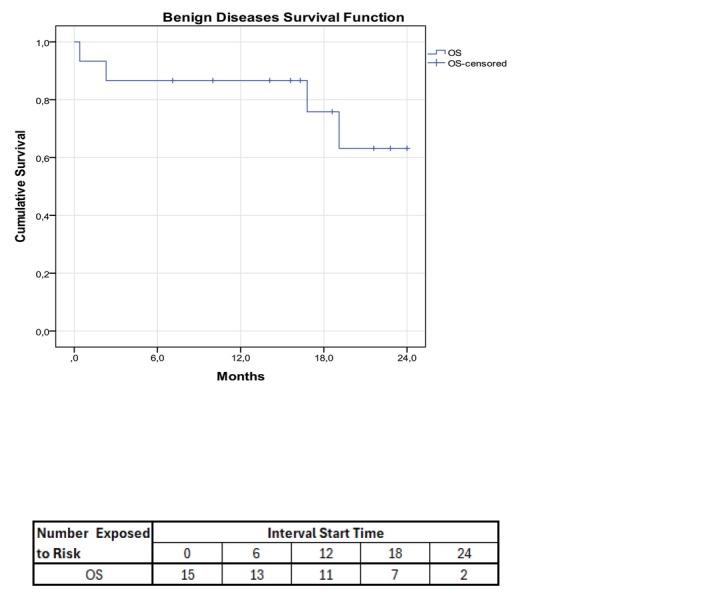
Overall Survival for benign diseases. 86.7% at 6 months, 86.7% at 12 months, and 73.3% at 24 months.

The Grade II acute GVHD in benign diseases was observed in 4/15 (26.6%) patients. Four of fourteen (28.6%) had moderate GVHD. Grades III–IV acute, and severe chronic GVHD were not observed. The 2‐year OS was 73.3% (95% CI, 51%–95.7%), Figure 1.

Twelve of the forty‐nine patients with malignant disease analyzed had relapsed at 24.5% (95% CI, 12.4%–36.5%). Ten relapses were acute leukemia, with a median time of 8.1 months, four ALL, and six AML. All this relapsed acute leukemia, despite new chemotherapy associated with DLI, had died, and the median survival time was 10.7 months (5.7–21.6). Two patients with HL relapsed. The first relapsed at 15.2 months and died from it at 20.2 months, and the other relapsed at 5.2 months and is alive with 22.3 months of follow‐up. One patient with MDS had autologous recovery and maintained pancytopenia with a follow‐up of 33.7 months.

**FIGURE 2:**
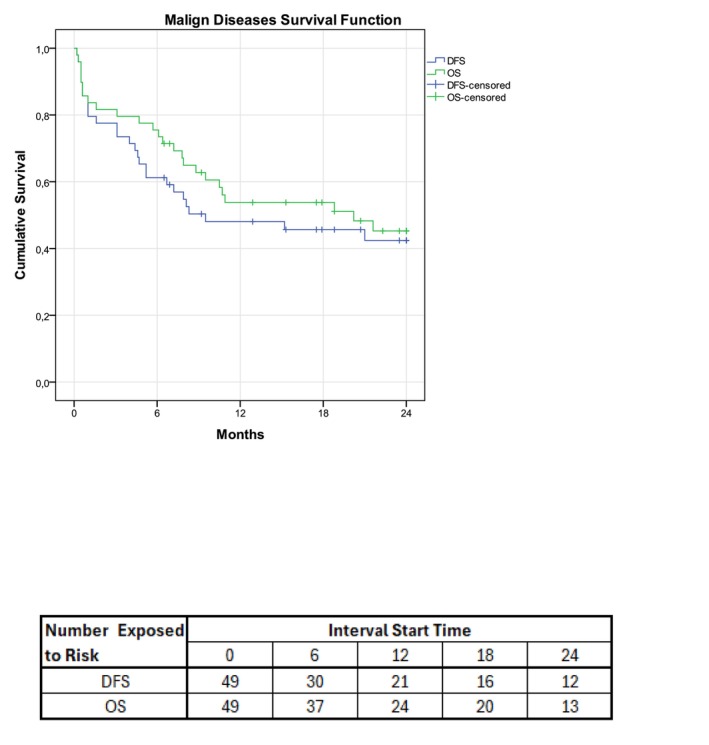
Disease‐free Survival and Overall Survival for malign diseases. Overall survival: 71.4% at 6 months, 54.4% at 12 months, and 49% at 24 months. Disease‐free survival: 59.2% at 6 months, 48.2% at 12 months, and 44.9% at 24 months.

Death concerning 100 days was in 5/57 after engraftment (8.8%). After 100 days, the death was related to relapse, observed in 64.7% (11/17; 95% CI, 42%–87.4%) patients in this period, Table 6. No variable was significant in the univariate analysis for relapsed. In malignant patients, the 2‐year relapse, DFS, and OS were 24.5% (95% CI, 12.4%–36.5%), 44.9% (95% CI, 31%–58.8%), and 49% (95% CI, 35%–63%), respectively. OS at 2 years for benign diseases was 73.3% (95% CI, 51%–95.7%), Figures 1 and 2. Acute III–IV and moderate/severe chronic GVHD were observed in 12.3% (95% CI, 3.8%–20.8%) and 21.2% (95% CI, 10.1%–32.3%), respectively, Table 2.

## 4 Discussion

While the initial Hid‐HCT with PT‐Cy regimen employed marrow BM grafts, many centers prefer PBSC. The classical Baltimore's group study with PT‐Cy with BM source observed graft failure occurred in 9 of 66 (13%) evaluable patients and was fatal in 1. The cumulative incidences of Grades II–IV and III–IV acute GVHD by Day 200 were 34% and 6%, respectively. There was a trend toward a lower risk of extensive chronic GVHD among recipients of 2 versus 1 dose of PT‐Cy (*p* = 0.05), the only difference between these groups. The cumulative incidences of NRM and relapse at 1 year were 15% and 51%, respectively. Actuarial OS and EFS 2‐year after transplantation were 36% and 26%, respectively [6].

In a prior large prospective study, PBSC has proven to cause a higher risk of chronic GVHD but lower engraftment failure in a population of mostly matched unrelated donor transplants who did not receive PT‐Cy [24]. Given the impact of PT‐Cy on lowering chronic GVHD rates, other investigators have evaluated PBSC with PT‐Cy versus bone marrow transplant with PT‐Cy. Bashey et al. compared 681 patients and found that bone marrow source resulted in significantly lower acute GVHD (25% vs. 42%) and chronic GVHD (20% vs. 41%), but higher relapse rates (45% vs. 28%) compared with PBSC. Engraftment, NRM, and OS were similar in the two groups. In subgroup analysis, they confirmed that the difference in relapse rates was found in patients with myeloid diseases but not lymphoma [7]. In a similar study of 86 patients, no differences were found in GVHD rates, but relapse was higher in the marrow group (58% vs. 24%) [26]. Though prospective data are needed, relapse rates may be improved with PBSC, while PT‐Cy may mitigate the risk of GVHD.

About the malignant diseases in this analysis, the 2‐year DFS and OS were 44.9% and 49%, and acute III–IV and moderate/severe chronic GVHD were observed in 12.3% and 21.2%, respectively. Xu et al. demonstrated the superiority of DSC over PBSC in acute leukemias with MAC and ATG. They observed a lower incidence of 2‐year NRM (62.5% ± 14.8% vs. 35.1% ± 5.1%; *p* = 0.014), higher OS (26.8% ± 12.3% vs. 43.2% ± 5.0%; *p* = 0.052) and DFS (26.8% ± 12.3% vs. 42.4% ± 5.0%; *p* = 0.071), respectively [27]. In 2019, the EBMT compared Hid‐HCT with RIC or MAC, in AML patients older than 45 years. RIC was performed in 539 patients, with 78% receiving PT‐Cy, and the source was PBSC in 60.3%. The 2‐year LFS, OS, acute GVHD III–IV, and chronic extensive GVHD were 41%, 44.1%, 10.5%, and 15%, respectively [28].

In 2016, the EBMT analyzed the intensity of conditioning in Hid‐HCT using PT‐Cy for acute leukemia. In patients with RIC, 65% received PBSC and 35% BM. Acute GVHD III–IV and chronic GvHD were observed in 10.9% and 24.7%, respectively. The LFS and OS at 2 years for patients in CR1, ≥ CR2, and active disease were 38.9% and 46.9%, 24.6% and 29.8%, 11.3% and 14.3%, respectively [29].

A meta‐analysis comparing PBSC and BM in Hid‐HCT with PT‐Cy in adult patients showed significantly elevated incidences of acute GVHD III–IV (OR = 1.741, 95% CI 1032–2938) and II–IV (OR = 1778, 95% CI 1314, 2406) and higher engrafting rates (OR = 1843, 95% CI 1066–3185) in the PBSC group. No significant difference was found in 2‐year relapse incidence, OS, and DFS. The pooled OS of single‐arm BM and PB studies were 0.543 and 0.591. The pooled incidence of DFS in BM and PBSC studies were 0.438 and 0.519 [30]. Hid‐HCT with ATG and DSC achieved similar results as identical allogeneic HCT in acute leukemias, MDS, and SAA [31]. In our group, the number of CD34^+^ cells infused with PBSC reduced the risk of disease progression. Maybe this finding could be explained by the larger number of T‐lymphocytes on the mobilized PBSC, which can induce a stronger graft‐versus‐tumor.

The Beijing Protocol for non‐malignant diseases such as SAA showed acute GVHD of 4% and 7.9%, extensive chronic GVHD of 4% and 5%, 3y OS of 84.6% and 89%, and 3y FFS of 84.6% and 86.8%, for adults and children/adolescents, respectively [32, 33]. Wang, Y. et al., in 756 Hid‐HCT with malignant and benign diseases using the Beijing Protocol, the 3‐year cumulative NRM was 18%, the 3‐year LFS was 68%, and 49% for low‐risk and high‐risk patients, respectively [34]. DeZern, A. E. et al. added ATG to the classic Baltimore Protocol for SAA in 37 patients and BM source. The cumulative incidence of grade II‐IV acute GVHD at Day 100 was 11%. The cumulative index of 2‐year chronic GVHD was 8% [35].

HCT‐CI ≥ 2 was observed in 33.7% and 17% of patients in two Brazilian publications of *Sistema Único de Saúde* (SUS) patients [36, 37]. In this survey, an HCT‐CI ≥ 2 was observed in 12.5% of the transplants and increased the death risk. In an analysis in four French centers with 223 patients, the impact of HCT‐CI in Hid‐HCT and PT‐Cy, with PBSC (*n* = 207) or BM (*n* = 8), did not observe statistical significance [38]. This study sample size was small to analyze OS for DRI. Although for Hid‐HCT, NMA with PT‐Cy, McCurdy, et al. observed three‐year OS 71%, 48%, and 35%, for low, intermediate, and high/very high risk, respectively [39].

Infection‐related mortality (IRM) was observed in 35% of cases in the series of 3394 allogeneic HCT in 2705 adults and 689 pediatrics in the Australian Registry, between 2013 and 2018. In adults, IRM was worse in the elderly, use of ATG or alemtuzumab, CMV serology positive in donor and negative recipient, and acute GVHD. In children, the risk was higher for ALL, unrelated to mismatch, and Hid‐HCT. Bacterial etiology was responsible for 49.3% of IRM in adults and the virus for 45% in children [40]. The *Grupo Español de Trasplante Hematopoyético*, in 236 Hid‐HCT, observed a 3‐year IRM of 19%. CMV infection was 69%. All these patients received PT‐Cy, 68% received RIC, and 32% MAC, 81% received PBSC, and 19% BM. IRM risk factors for IRM were age over 50 years, lymphoid neoplasms, and GVHD III‐IV. The etiology of the IRM was bacterial in 51%, most of them Gram‐negative bacteria [41]. In these two publications, from Australian and Spanish groups, infections and colonization before transplants are not analyzed.

In our series, there were 13 deaths due to bacterial infection, six before and seven after engraftment, in the 64 Hid‐HCT performed (20.3%). It is worth noting that 31.5% of the patients were colonized by CRGNB and/or VRE. Colonization of patients by CRGNB led to early mortality that compromised the engraftment. It is important to note that in Brazil, the ceftazidime/avibactam approval of use occurred in June 2018. During the study time, the drug was not available in these institutions. Comparing the results observed with IRM in the literature, we can suggest a benefit with the use of DSC in Hid‐HCT with PT‐Cy, despite the quantitative limitation of our series, the lack of population data on colonization, and previous infections in these other studies. It is necessary to highlight the prior swabs to assess colonization by resistant bacteria. CRGNB was significant in univariate analysis for DFS. CMV infection was 45.6%, comparable to the results observed in the cited publications. The CIBMTR analyzing Hid‐HCT with PT‐Cy from 2012 to 2017 observed 42% CMV infection. In identical allogeneic HCT use of PT‐Cy, there were also more infections than allogeneic without PT‐Cy, 37% and 23%, respectively. PT‐Cy, regardless of the donor, was associated with a higher incidence of CMV infection, increasing the risk of seropositivity, regardless of the donor [42].

## 5 Conclusion

The results observed with DSC and PT‐Cy in Hid‐HCT in this group of patients are difficult to compare with previous publications. An interesting view in this survey was the HCT‐CI that predicted DFS and OS. The characteristics of the analyzed population, infectious data, and conditioning intensity are preliminary for further studies to consolidate the results.

## Author Contributions

**Ana Marcela Rojas Fonseca‐Hial:** data curation (lead), formal analysis (lead), investigation (lead), methodology (equal), project administration (equal), resources (equal), supervision (equal), validation (equal), visualization (equal), writing – original draft (lead), writing – review and editing (lead). **Katya Parisio:** data curation (supporting), formal analysis (equal), validation (equal). **Jose Salvador Rodrigues de Oliveira:** conceptualization (lead), data curation (equal), formal analysis (equal), investigation (lead), methodology (lead), project administration (lead), resources (lead), supervision (lead), validation (lead), visualization (equal), writing – original draft (equal), writing – review and editing (lead).

## Consent

Because of the retrospective and anonymous character of this study, the need for informed consent was waived by the institutional review board and was granted by the “*Federal University of Sao Paulo Ethics Committee*” for this purpose.

## Conflicts of Interest

The authors declare no conflicts of interest.

## Data Availability

The authors confirm that the data supporting the findings of this study are available from the corresponding author, upon reasonable request due to privacy/ethical restrictions.
